# Review of interactions between phosphorus and arsenic in soils from four case studies

**DOI:** 10.1186/s12932-018-0055-6

**Published:** 2018-04-02

**Authors:** Daniel G. Strawn

**Affiliations:** Department of Soil and Water Systems, University of Idaho, Moscow, ID 83844-2340 USA

## Abstract

Arsenic is a non-essential element that poses risks in many environments, including soil, groundwater, and surface water. Insights into the environmental biogeochemistry of As can be gained by comparing As and P reaction processes. Arsenic and P are chemical analogues, and it is proposed that they have similar chemical behaviors in environmental systems. However some chemical properties of As and P are distinct, such as redox reactions, causing the biogeochemical behavior of the two elements to differ. In the environment, As occurs as either As(V) or As(III) oxyanions (e.g., AsO_4_^3−^ or AsO_3_^3−^). In contrast, P occurs predominantly as oxidation state five plus; most commonly as the orthophosphate ion (PO_4_^3−^). In this paper, data from four published case studies are presented with a focus on P and As distribution and speciation in soil. The goal is show how analyzing P chemistry in soils can provide greater insights into As reaction processes in soils. The case studies discussed include: (1) soil developed from shale parent material, (2) mine-waste impacted wetland soils, (3) phosphate-amended contaminated soil, and (4) plants grown in biochar-amended, mine-contaminated soil. Data show that while P and As have competitive reactions in soils, in most natural systems they have distinct biogeochemical processes that create differing mobility and bioavailability. These processes include redox reactions and rhizosphere processes that affect As bioavailability. Results from these case studies are used as examples to illustrate how studying P and As together allows for enhanced interpretation of As biogeochemical processes in soils.

## Introduction

Arsenic is a naturally occurring element in soils, sediments, and the subsurface. It occurs in surface water, plants, and groundwater. In many environments, As poses risks to humans or animals because of elevated concentrations in water or plant samples [1]. Elevated arsenic concentrations are especially prevalent in mine-impacted environments. The environmental risks are influenced by management of soils, sediments, groundwater, surface water, and ecosystems. Due to the active biogeochemical cycle of As, knowledge of its species and reactions in natural and managed systems is required to reduce contamination risks.

**Fig. 1:**
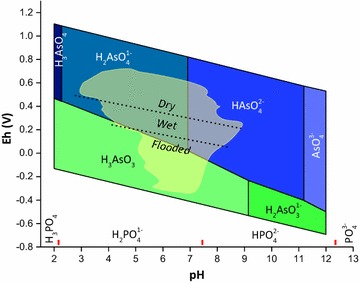
Thermodynamic prediction of arsenic and phosphorus speciation (red marks) in aqueous systems (T = 25 °C, 1 atm.) with overlay of domain of 414 soil Eh and pH measurement reported in Becking et al. [3]. Dash lines designate soil water status reported by Becking et al. [3]. Wet soils refer to soils that are seasonally flooded. Upper and lower redox boundaries delineate stability lines for water. Arsenic predominance diagram adapted from Smedley [2]

In the environment, As occurs as organic and inorganic compounds. The two most prevalent oxidation states in the environment are As(III) and As(V), which occur as oxyanions arsenite (AsO_3_^3−^) or arsenate (AsO_4_^3−^) [2]. Figure 1 shows thermodynamic modeling of the redox predominance diagram for As and compares this to soil moisture status. The diagram shows that in most unsaturated soils, As should exist as arsenate, but in inundated soils, arsenite may become the predominant species if the system becomes reducing enough. The measured Eh–pH domain observed for 414 soils from around the world is shown in Fig. 1 [3], which shows that arsenite is thermodynamically stable in soils that undergo seasonal flooding (wet) and soils that are permanently flooded. While instructive, redox predominance diagrams are based on equilibrium, and do not consider kinetics, nor other chemical species that occur in natural environments that affect As reaction processes. These factors make quantitative predictions of As species using thermodynamics and pure systems inaccurate.

Arsenite has been observed in water saturated soil environments, groundwater, and in river, lake and marine sediments [4–7]. In rice paddies, arsenite is an important species controlling As bioavailability [8, 9]. At low to neutral pH, arsenite has less adsorption affinity on soil mineral surfaces than arsenate, and thus greater biological availability [10–12]. Rukh et al. [13] measured arsenite and arsenate adsorption capacity of four soils (pH 7–8.4) and observed that two to five times more arsenate was adsorbed on the soils than arsenite. Manning and Goldberg [12] observed that more arsenate than arsenite was adsorbed on three California soils (pH 5.7–7.1); but they noted that if the soils were incubated in alkaline pH solutions, arsenite adsorption predominated. Surface complexation modeling by Gustaffson and Bhattacharya [14] suggests that arsenate forms stronger surface complexes on oxide surfaces compared to arsenite. As a result of the differing adsorption behavior of arsenite and arsenate on soils, the solubility of arsenic in soil pore water is dependent on its oxidation state. Because arsenic undergoes redox transitions in low oxygen environments, redox driven solubility is a major factor affecting the fate of arsenic in wetlands, sediments and inundated soils.

Arsenic belongs to periodic group 15. Arsenate (As(V)) has an empty outer electron shell, missing five valence electrons ([Ar]3d^10^4s^0^4p^0^). Phosphorus (P) also belongs to periodic group 15, and, like As(V), P predominantly occurs as oxidation state five plus species in the environment, with an electron configuration of [Ne]3s^0^3p^0^. In aqueous environments, As(V) and P(V) exist as the oxyanions arsenate (AsO_4_^3−^) and phosphate (PO_4_^3−^), surrounded by four oxygen atoms in tetrahedral coordination; one oxygen attached to As or P via a double bonded atom and three acidic oxygens attached via single bonds. Because of their similar chemical speciation, phosphate and arsenate are considered chemical analogues, implying that they can substitute for each other in chemical reactions. In biogeochemical reactions, this is often observed, including adsorption/desorption reactions [15–20], precipitation/dissolution reactions [21, 22], and competitive absorption in plant and microbial systems [23–27]. O’Reilly et al. [28] measured arsenate desorption using phosphate solutions and observed that only 35% of the adsorbed arsenate was desorbed from goethite, suggesting that arsenate bonds on iron oxide are stronger than phosphate bonds. In organisms, the metabolic substitution of arsenate for phosphate may be a toxicity mechanism [29, 30]. In aqueous solution, arsenate and phosphate occur as triprotic arsenic and phosphoric acids that have similar deprotonation constants (Table 1 and Fig. 1).

**Table 1:** Chemical properties of phosphorus and arsenic

	Stable oxidation states in environment	Acidity constants	Crystal radius^a^
Phosphorus	P^5+^ ([Ne]3s^0^3p^0^)	H_3_PO_4_: 10^−2.15^, 10^−7.2^, 10^−12.35^	Phosphate 31 pm
Arsenic	As^3+^ ([Ar]3d^10^4s^2^4p^0^) As^5+^ ([Ar]3d^10^4s^0^4p^0^)	H_3_AsO_3_: 10^−9.2^ H_3_AsO_4_: 10^−2.19^, 10^−6.94^, 10^−11.5^	Arsenate 47.5 pm

^a^[112]

Competitive biogeochemical reaction processes between phosphate and arsenate control the fate and bioavailability of arsenic in the environment. However, redox reactions of arsenic in the environment create distinct biogeochemical reactions that do not occur for P. For example, under reducing conditions, such as in rice paddies, arsenate is reduced to arsenite [8, 9], which does not adsorb as strongly to soil particles as arsenate (especially at low to neutral pH), and thus is more soluble and mobile. The redox driven mobility creates distinct distributions of arsenic in soils and sediments that undergo redox fluctuations. Phosphorus, on the other hand, is not directly affected by redox changes.

Numerous studies have investigated the effects of redox on As distributions in wetland and sediment environments [31–33]. Studies on competitive reactions between phosphate and arsenate for uptake by plants are also numerous [27, 34, 35]. However, few studies have done a comparative analysis of As and P biogeochemical cycling in soils. In this paper, data from four papers are presented to investigate As and P interactions in soils. A new, enhanced interpretation of the data is done to infer interactions between P and As in the soils. It is shown that by evaluating P distribution and availability in soils, greater insight into the biogeochemical processes controlling As mobility and distribution in natural systems can be deduced.

## Methods

Data presented in this paper were gleaned from four published papers. Detailed methods are published in the corresponding papers. A summary of the methods used in the four different experimental systems investigated is provided below.

### As and P in soil formed from shale parent material

Strawn et al. [36] studied the distribution of As in soil in the Panoche Hills on the Eastern side of the California Coast Range. The parent material for the soil is a shale rock. Due to mass wasting, partially weathered shale parent material was present in the A horizon. This soil is classified as an Ultic Haploxeralf. The soils have a relatively high salt content and low pH (pH = 4.0), indicative of the oxidation of pyritic mineral inclusions in the shale materials (sulfuricization) [37]. Thin sections of the soils were prepared from intact cores of the A horizon. Elemental distribution of Fe, S, Ca, K, As, P and Se were collected on an X-ray microprobe beamline (Beamline 10.3.2 Advanced Light Source, Berkeley, California). After mapping, As K-edge X-ray absorption near edge structure (XANES) spectra were collected from points of interest to determine the As oxidation state.

### As and P distribution in a mine-waste contaminated wetland soil

Strawn et al. [38] studied the distribution of As and P in a mine-waste contaminated wetland in Black Rock Slough in the Coeur d’Alene River (CdA) floodplain located in Northern Idaho. The wetland soils in the CdA River Basin have elevated concentrations of As, Cd, Pb and Zn due to more than a century of mining activities in the watershed that has redistributed the mining and milling materials throughout the floodplain [39–41]. The soils in the floodplain wetlands are classified as Fluvaquents in Soil Taxonomy [42]. A full profile description at this sampling site is reported in Hickey et al. [43]. Four sampling points along an 80-m long transect were sampled. The elevation change of the transect is 1 m. Elemental content of bulk soils and redox masses were measured by digesting the samples in aqua regia and HF solution (EPA Method 3052 [44]). The samples were then analyzed for As, Cd, Fe, Mn, P, Pb, and Zn on a ICP-AES standardized using certified standards.

### Effects of P remediation of Pb contaminated soil on As availability

Soil samples for the phosphate amendment remediation trials were collected from the Black Rock Slough located on the floodplain adjacent to the CdA River in Kootenai County, Idaho [45]. Four samples were collected and composited. The soils at the sites are floodplain soils heavily influenced by mine tailing and ore processing runoff that was transported and deposited in the lower CDA floodplain (described above).

Phosphorus sorption isotherm experiments were carried out on the composite soil sample using spiked P concentrations from 0.098 to 198 mg L^−1^ P. The suspensions were placed on a reciprocal shaker for 24 h and subsequently centrifuged and filtered through a 0.45 µm PES filter membrane (Millipore Inc., Ann Arbor, MI). Following application of the phosphate amendments, Bray extractions [46] and TCLP extractions [47] were performed on the soil paste from the isotherm experiment to assess potentially leachable P, As and Pb after amendment of the soil with the P. All solutions were analyzed on an ICP-AES spectrometer using certified standards.

### Use of biochar amendment in contaminated soils to reduce bioavailability of As to plants

Strawn et al. [48] investigated the effects of biochar on As bioavailability using Mountain Brome (*Bromus marginatus*) plants grown in greenhouse trials with soil from the Stibnite Mine site in central Idaho (14 miles east of the town of Yellow Pine, Idaho). The goal of the study was to see how biochar affected As bioavailability in the soils. Using plant uptake is a direct indicator of As bioavailability. Mountain Brome is an important native grass species in North America that is included in many reclamation seed mixes. A composite soil sample was collected from thee points on a tailings pile at the Stibnite Mine. X-ray absorption near-edge structure (XANES) spectra were measured to provide a molecular scale interpretation of As in the soils, and determine if biochar amendment caused changes in the As oxidation state. Biochar used in this experiment is a byproduct of a boiler with lumber milling waste feedstock that uses variable incineration temperatures.

Biochar was applied to the soils at a rate of 10% by mass. Mountain Brome grass seed (Granite Seed Company, Lehi, UT) was grown for ~ 12 weeks. The experimental design included five replicates of biochar and non-biochar amended soils. At harvest, above ground plant material was cut, and roots were isolated from the soil and carefully washed in DI water to remove all visible soil. Replicate subsamples of dried biomass were digested in hot aqua regia using the EPA 3050B digestion method [49], and analyzed on an ICP-AES to determine As and P content.

Arsenic K-edge XANES spectra from soils collected from the greenhouse pots were collected on a bending magnet beamline (Sector 20) at the Advanced Photon Source (Chicago, IL) in fluorescence detection mode. The samples were scanned in the energy range of 11,800–11,950 eV.

## Results and discussion

### As and P in soil derived from shale

**Fig. 2:**
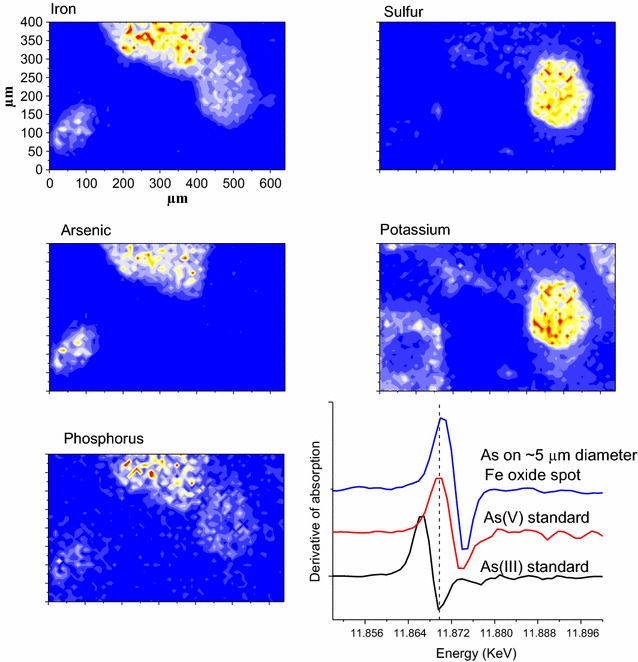
Arsenic XANES spectra and elemental distribution of Fe, S, As, K and P in a soil thin section made from the A horizon of a soil that developed on a pyrite-containing shale in the Panoche Hills of California (Data are from Strawn et al. [36])

Within the soil thin section, submillimeter regions containing red or orange iron oxides (Fig. 2), and bright yellow jarosite were observed. Elemental mapping suggest that these domains are iron oxide and jarosite (Fig. 2). XAFS spectral analysis of the regions of interest confirmed that the mineral species are ferrihydrite and jarosite [50]. The elemental distribution of P in the soil thin section shows the highest P concentrations in the jarosite and iron oxide aggregates, with a direct correlation between Fe and P (Fig. 2). In contrast to P, As is associated only with the iron oxide aggregates and not the jarosite aggregates. XANES analysis of the As in the iron oxide regions indicated it was present as As(V) oxidation state species. Kendall et al. [51] conducted dissolution experiments of synthetic arsenic-substituted jarosite and observed that secondary reaction products were arsenic adsorbed on iron oxides instead of jarosite. They proposed that the bond between arsenate and iron is stronger than the iron-sulfate bond that typically occurs in jarosite, and thus the arsenate destabilizes the jarosite, favoring formation of an iron oxide with arsenate adsorbed. In contrast, Kato et al. [52] proposed that the bond length between P and oxygen is within the crystal lattice dimensions of the tetrahedral positions in jarosite, although it likely creates some strain because it is greater than the ideal sulfur to oxygen bond length. The larger ionic radius of As compared to P (Table 1) apparently causes greater strain in the jarosite structure than P, thus making As-substituted jarosite less stable and less likely to occur in nature.

Arsenic and P are both known to adsorb onto iron oxides through inner-sphere bonds [53, 54], and ferrihydrite has relatively high surface adsorption sites compared to other iron oxides. Thus both As and P can adsorb on the surfaces of the ferrihydrite minerals in the Panoche Hill’s soils. In a natural system at low pH, such as the soil in this study (soil pH = 4.0), there are excess number of binding sites on ferrihydrite minerals for both As and P (molar Fe:P:As = 550:20:0.19), thus competitive adsorption between As and P may not be significant. Dudas et al. [55] determined that the total As in mineral separates from the B and C horizons of an acid-sulfate soil that formed from shale materials in a forest in Alberta, Canada were concentrated in the iron oxide fraction by 10–20 times the concentrations occurring in the jarosite minerals separated from the soils, and at least 10 times the bulk soil concentration. In weathered pyritic mine tailings, Foster et al. [56] observed that As occurred in either scorodite or adsorbed on the surfaces of iron and aluminum oxides. Savage et al. [57] observed that in weathered pyritic mine tailings, As was either adsorbed on the surfaces of iron oxides, or coprecipitated with iron sulfate minerals. Lumsdon et al. [58] measured arsenopyrite/pyrite particle weathering in laboratory simulations and observed that dissolved As concentrations in solutions were the lowest when the weathering products had advanced past jarosite to amorphous iron oxides, suggesting a greater degree of As adsorption on the iron oxides than jarosite.

Results from the Panoche Hills soil show microscale evidence that iron oxides preferentially accumulate As compared to jarosite. By comparing the micro-scale P distribution in the soil, insights into reaction processes occurring during soil weathering were made. Such results provide new knowledge of the fate of As during soil formation.

### As and P distribution in a mine-waste contaminated wetland soil

**Fig. 3:**
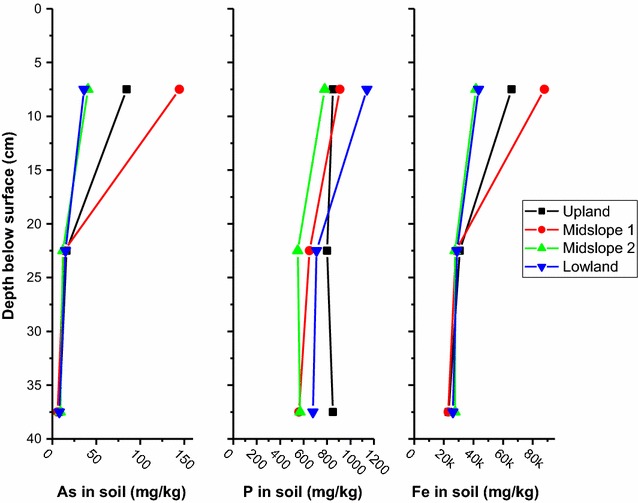
Total As, P and Fe content in wetland soils as a function of depth in soils at a mine-contaminated site in Coeur d’Alene River Basin, Idaho. Four sites were sampled along an 80-m long transect with an elevation change of 1.1 m (Data are from Strawn et al. [38])

The soils in the Black Rock Slough wetland in CdA have elevated concentrations of As (Fig. 3). Surface horizons of the upslope soils have greater As concentrations than surface horizons of the lowland soils. This elevation-dependent trend is also observed for Fe (Fig. 3) and Mn concentrations [43]. Phosphorus does not show a concentration gradient in the soils (Fig. 2). Baker et al. [59] also reported an enrichment of As and Fe in the surface of soils from the Black Rock Slough, and a lack of concentration gradient for P. The enrichment of As and Fe in the surface horizons in the Black Rock Slough soils suggests that in the upland soils, where soil redox potentials undergo the most dramatic changes due to the seasonal water table, As is undergoing redox-controlled dissolution or desorption reactions. The reactions are driven by biogeochemical processes that reduce both As and Fe in the soils. Reactions include reduction of arsenate to the more soluble arsenite, and reductive-dissolution of iron oxides $\left( {{\text{FeIII-oxide}}\,\left( {\text{s}} \right) \to {\text{Fe}}^{ 2+ } \left( {\text{aq}} \right)} \right).$ Haus et al. [60] and Toevs et al. [61] also observed As concentration gradients in CdA River Basin lateral lake sediments that were subject to large fluctuations in water levels (i.e., redox changes), while As concentration profiles were not affected by redox promoted translocation in sediments without redox fluctuations.

**Fig. 4:**
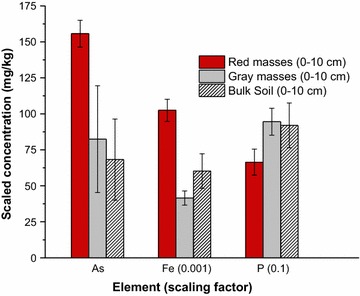
Average total As, P and Fe content in wetland soil (n = 4) isolates from a mine-contaminated site in Coeur d’Alene River Basin, Idaho. Red masses refer to soil fractions isolated from the soil that are elevated in iron oxides. Gray masses refer to soil fractions isolated from the soil that are depleted in iron oxides. Error bars are standard deviations (Data are from Strawn et al. [38])

Iron-enriched redoximorphic masses in the soils had up to 1.5 times the As concentrations compared to non-iron enriched (gray) masses (Fig. 4). The masses range in size from a few millimeters to several centimeters [43]. Their occurrence is a direct result of reductive dissolution and translocation of Fe to zones within the soil that are more oxidizing. Phosphorus concentrations were less in the iron enriched soil redox masses than in the gray redox masses, which is the opposite trend compared to As; in fact, the iron rich masses are also depleted in P compared to the bulk soils.

Comparison of As and P data suggests that there are distinct biogeochemical process separating the elements in the soils. Although P does not get reduced in flooded soils, reductive dissolution of the Fe minerals that P is associated with may release it to the soil solution. Some research has shown increased P solubilization in reduced soils, while other research shows that reducing conditions promote decreased P solubilization [62–64]. An important factor in As and P release under reducing conditions is that some ferric iron is not reduced. This may be because the Fe(III) is located in microsites that are small oxic zones within the soil [65, 66], or because of slow redox dissolution reactions [32], or because of excess Fe(III) reactant. The presence of Fe(III) oxides in reduced soils will adsorb phosphate, even when they are present in small amounts because they have high surface area and high adsorption capacities at the low pH of the Black Rock Slough soils (pH = 3.9–5.1). In contrast to P, under reducing conditions, arsenate is reduced to arsenite, which is more soluble and mobile, and thus, at low pH conditions, not as likely to adsorb to iron oxides that persist under reducing conditions.

**Fig. 5:**
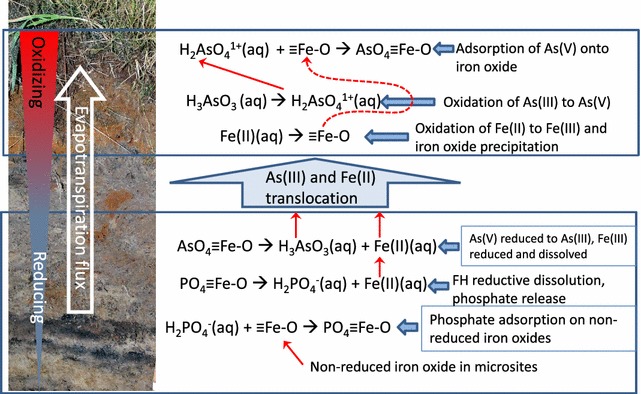
Proposed biogeochemical processes affecting Fe, As, and P distributions in wetland soils (Adapted from Strawn et al. [38])

Distributions of As and P concentrations in the soil at the Black Rock Slough wetland are influenced by the distinct redox behaviors of these two elements. Specifically, As is translocated with Fe during redox-driven fluxes, whereas phosphate is not affected by redox. Thus, in soils where redox is constantly changing, a depth gradient and redoximorphic concentrations with Fe and As are created, while P concentrations in the soil matrix do not change, likely because they are adsorbed onto non-reduced iron oxides. An illustration of this process is shown in Fig. 5. Redox controlled As movement and P distribution process have important implications on the distribution and availability of As in the environment, and should be included in designing best management strategies of contaminated environments. For example, managing contaminated wetland soils to minimize cyclic redox conditions will prevent redox-driven translocation of As from the lower profile to the surface.

### Effects of P remediation of Pb contaminated soil on As availability

Contamination of soil by mine tailings often results in occurrence of multiple contaminants being present. In soils in the Bunker Hill Superfund site located in the Coeur d’Alene River watershed, Zn, Pb, As, and Cd have contaminated thousands of acres of soil and sediment [40, 67, 68]. To remediate the soils in the CdA Basin, in situ remediation strategies using phosphate application are being considered. Amendment with phosphate decreases Pb solubility in soils by promoting the formation of lead phosphate minerals such as pyromorphite (Pb_5_(PO_4_)_3_Cl) that have low solubility [69–73]. However, use of phosphate as a remediation amendment for soils may pose risks for off-site transport of the P to surface waters, creating an increased risk of eutrophication in lakes and rivers [74–76]. In addition, phosphate amendment may cause an increase in As mobility and bioavailability through competitive adsorption reactions. In this study, the effects of phosphate amendment of mine-waste contaminated soils on Pb immobilization and As mobilization were investigated [45].

**Fig. 6:**
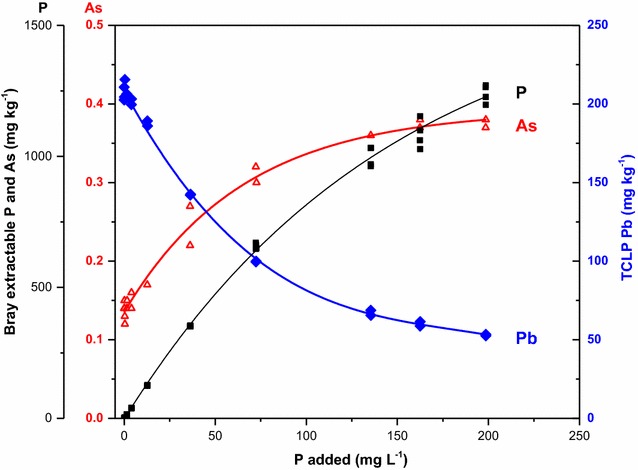
Effect of phosphate amendment on extractable As and P concentrations using Bray extraction solution, and extractable Pb concentrations using TCLP extraction solution. Soils are mine-contaminated wetland soils from Coeur d’Alene River Basin, Idaho (Data are from Osborne et al. [45])

At the lowest concentrations of P amendment, nearly all of the P added to the soil suspensions adsorbed onto the soil surfaces. With increasing concentrations of added P, adsorption sites became saturated; maximum P adsorption was ~ 1400 mg kg^−1^ (Fig. 6). Phosphorus extractability increased as the amount of P added to the soil suspensions increased.

TCLP extraction of Pb from soils provides a relative measure of the Pb availability for leaching or uptake by an organism, and thus can be used to assess the amount of Pb immobilized by P amendment [70, 77, 78]. For soils, TCLP extractable Pb decreased with increasing initial P added to the soil suspension (Fig. 6). TCLP extractable Pb appears to become asymptotic as P amendment rate increases, suggesting that there is a maximum immobilization potential of P amendment under the conditions of these experiments, and that higher P amendment rates will not decrease TCLP extractable Pb.

Increasing the amount of phosphate amendment caused Bray extractable As to increase (Fig. 6). Increases in extractable As after P remediation of soils have been reported in other studies [79–81]. In the contaminated soil sample, at the highest phosphate amendment rate (198 mg P L^−1^), Bray extractable As was approximately three times greater than in the non-phosphate amended soil (Fig. 6). The CdA soils contain high concentrations of iron oxides (total Fe 6% by weight), which is a sink for added P and As, especially at the low soil pH of the CdA soils (pH = 4.8). However, despite the high concentrations of iron oxides, the Olsen bicarbonate extractable As increased, suggesting that the P is saturating the adsorption capacity. Potential solubilized organic matter from the phosphate amendment may also be competing for the adsorption sites and increasing the As extractability.

Results show that use of phosphate to immobilize Pb in contaminated soils is effective. However, a negative side effect is the increased availability of As due to the competitive adsorption reactions between phosphate and arsenate. Because of the increased As solubilization from phosphate amendment, this remediation strategy may not be feasible for soils contaminated with both Pb and As. The feasibility of the remediation needs to be considered on a case-by-case basis because differing soil properties will affect the competitive nature of P and As adsorption and release.

### Use of biochar amendment in contaminated soils to reduce bioavailability of As to plants

Biochar amendment of contaminated soils has been shown to reduce contaminant mobility and bioavailability, while increasing success of revegetation [82–88]. In this study, the effects of biochar amendment on availability of As from soils for plant uptake were studied [48]. The soils are from the Stibnite mining area in central Idaho. After more than a century of mining activity for antimony, gold, silver, and tungsten, large waste piles containing high concentrations of As occur throughout the site [89–91]. To reduce As transport and leaching, and improve soil properties for plant growth, biochar amendment is being investigated as a possible remediation technology.

The average As concentration of the soil is 3541 mg kg^−1^. Average P concentration is 1310 mg kg^−1^. Soil pH is 8.25, which promotes less arsenate adsorption on iron oxides in soils than would occur if the soils had lower pH conditions.

**Fig. 7:**
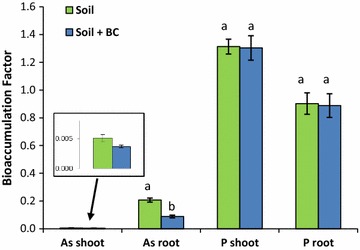
Arsenic and P concentrations in plant tissue grown in mine-waste contaminated soils amended with biochar (BC) and unamended soils. Soils are from the Stibnite mine near Yellow Pine, Idaho. Error bars are one standard error (Adapted from Strawn et al. [48])

Arsenic concentrations in the plant tissue grown in both the biochar-amended and unamended Stibnite mine soils were more than 100 times greater (11.8–659 mg kg^−1^) than in typical plants grown in non-contaminated soils (0.1 mg kg^−1^, d.w. [92]). Bioaccumulation factors (BAF) for As and P were calculated by dividing element concentrations in plant tissue by total soil concentrations. In both amended and unamended soils, the root As BAFs were much greater than the shoot As BAFs (Fig. 7). Plant root As BAFs in the unamended soils were much greater than As concentrations in the plant roots grown in the biochar-amended soils. A similar trend occurred in the shoots, although, due to one outlier, the difference was not significant at 0.05 level.

Beesley et al. [93] observed that the addition of biochar increased the water leachable fraction of As. But in another study, Beesley et al. [94] observed that biochar amendment of an As contaminated soil increased pore water As concentrations and decreased tomato plant tissue As concentrations. Hartley et al. [95] observed that the addition of biochar to As contaminated soils had little effect on As uptake by Miscanthus (Miscanthus × giganteus). Gregory et al. [96] observed that biochar amendment to an As contaminated soil increased arsenic concentration in ryegrass. Namgay et al. [97] observed that biochar amendment decreased As uptake in maize shoots. The varying and contrasting As uptake behavior by plants from these studies when the soils were amended with biochar suggests that the biochemistry between biochar, soil, and plants are specific to the types of these components in the system being studied.

Phosphorus BAFs in roots were less than in the shoots (Fig. 7), which is opposite the As trend. Biochar amendment did not affect the P availability from soils for uptake in the plant tissues. Plant tissue As concentrations were ~ 100 times less than P, despite the much greater As concentrations in the soil compared to P concentrations (~ 3×). This suggests that Mountain Brome selectively absorbs P compared to As, possibly because either, (1) As is less soluble in the soils than P, (2) microbial and rhizosphere processes in the soils alter the As and P bioavailability [98, 99]; (3) specific plant protection mechanisms to resist As absorption [100, 101]; or (4) plant [102, 103] or soil microorganisms [104] volatilization of As make it less bioavailable (these processes are reviewed in Zhao et al. [105]). In most soil environments, except in flooded soils [106], volatilization is not a major process [107]. This study was not designed to assess plant rhizosphere biochemistry, but only evaluate how biochar affects bioavailability of soil As.

**Fig. 8:**
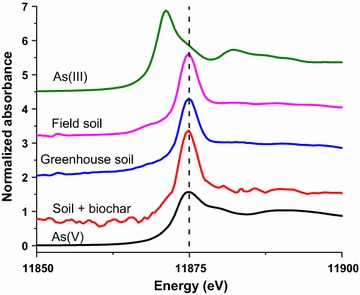
X-ray absorption near edge structure (XANES) spectra of As K-edge from samples incubated with and without biochar in greenhouse soils and field soils (unamended). K-edge XANES spectra for sodium arsenate (As(V)) and sodium arsenite (As(III)) standards are also included as oxidation state edge-energy references (Adapted from Strawn et al. [48])

XANES spectra in all soils have peaks at 11,875 eV, (Fig. 8), indicating that the As oxidation state in the soils is As(V), which occurs as the oxyanion arsenate. Arsenate adsorbs strongly to soil mineral surfaces, such as iron oxides. There are no differences in the As XANES spectra between the biochar-amended and unamended soils, suggesting no change in As oxidation state in the soils.

Biochar amendment of the soil decreased bioavailability of As from the soils as assessed by Mt. Brome plants, but did not change phosphate uptake (Fig. 7). This suggests that biochar is changing arsenate speciation in the soils, but is not affecting the phosphate. Biochar amendment can change As plant bioavailability by changes in soil pH, increase in reactive surfaces to adsorb As, stimulating microbiological activity that can change As speciation, or by introducing available phosphate that competes for adsorption sites with arsenate. There was only a small change in pH from biochar amendment (~ 0.4 pH units), thus this is not a factor affecting As bioavailability. The lack of change in As oxidation state upon biochar amendment (Fig. 8), suggests that As reduction was not a factor affecting bioavailability.

Mountain Brome may have mycorrhizae that affects As uptake [108, 109]. Mycorrhizal roots take up P either directly through the root, or mediated pathways involving the mycorrhizae. The direct P pathway can take up As and P, while the mediated pathway downregulates the As uptake pathway and facilitates increased P uptake [110]. Biochar may facilitate mycorrhizal colonization of the plant roots [111], thus causing increased selectivity for P in the mediated P uptake pathway. This study was not designed to assess these factors, however, the results of decreased As bioavailability in the biochar-amended soils suggest that biochar is either changing As and P availability in the rhizosphere, or causing a change in the plant biochemistry, such as mycorrhizal associations. Additional research should be done to link plant biochemistry with As bioavailability in biochar-amended soils.

Based on the findings of this study, arsenate in the Stibnite soil does not behave the same as phosphate with respect to plant availability and biochar amendment. Biochar amendment did not impact P uptake from the contaminated soils, but did significantly decreases As uptake by Mountain Brome. By comparing bioavailability of As and P in biochar and non-biochar amended soils, important relations between soil and plant roots have been revealed.

## Integration of As and P biogeochemical processes in soils from case studies

In this paper, a comparative analysis of As and P in several different environmental systems was used to gain insight into how biogeochemical processes affect As mobility and bioavailability. Although As and P are chemical analogues, in the environment they have distinct biogeochemical reactions. In soils formed from weathered shale, As preferentially associates with iron oxides, while P associates with both iron oxides and jarosite minerals. This may be explained by differences in the ionic radius of As and P that lead to different adsorption strengths of arsenate vs phosphate. In wetland soil environments, comparison of As and P suggested that reduction of arsenate to arsenite facilitated translocation of As to the surface horizons of soils, while P was not impacted by redox cycling occurring in the wetland soils. In Pb-contaminated soils with As present as a co-contaminant, it was shown that phosphate amendment immobilizes the Pb, but via competitive adsorption reactions, increases As availability. In As-contaminated soils, biochar amendment decreased As bioavailability but not P; possibly by some biologically-mediated processes promoted by the biochar that allowed for selective uptake P over As by Mountain Brome plants.

The examples of As and P distribution and speciation in soils and changes in bioavailability from the four case studies show that, although As and P are chemically similar, they have unique biogeochemistry that can be leveraged to provide added knowledge of processes controlling the fate of As in the environment.
